# Supplementary material for: GaNDLF: the generally nuanced deep learning framework for scalable end-to-end clinical workflows
Source: Commun Eng. 2023 May 16;2:23. doi: 10.1038/s44172-023-00066-3 (PMC10956028; doi:10.1038/s44172-023-00066-3)
Supplement: Supplementary file 2 — Supplementary Information [file 44172_2023_66_MOESM2_ESM.pdf]

Supplementary Notes 1: Details of Data Augmentation

Following up on the Data Augmentation sub-section in Methods, this section contains a tabular illustration of *some* of the data augmentation techniques integrated into GaNDLF. The variety showcased here highlights the built-in flexibility of the entire framework.

Supplementary Table 1: All available data augmentations provided in GaNDLF.

Type	Augmentation	Description of Specific Application
Spatial	Affine	Random affine transformations
	Elastic	Dense random elastic deformations
	Flipping	Reversal of the order of elements in an image along the given axes
	Rotation	Rigid rotations of 90 or 180 degrees across the specified axes
	Anisotropic	Down-sample and up-sample images along the provided axes
Intensity	Blur	Blurring using a random-sized Gaussian filter
	Noise	Gaussian noise with random parameters
	Gamma	Random change of contrast by raising values to the power of γ
MRI Space	Bias field	Random MRI bias field artifact
	MRI motion	Random motion artifact
	Ghosting	Random ghosting artifact
	Spike	Random spike artifacts

Supplementary Methods: Network Architectures

GaNDLF seeks to provide both well-established and state-of-the-art network architectures showing promise in the field of healthcare. Since the literature on novel network architectures is continuously being expanded, at the time of publication the following list/table of architectures are offered by GaNDLF, the topologies of which are shown in the Methods section. Detailed description of each architecture is provided in the Supplementary Material.

- **UNet**: The UNet with (**ResUNet**) and without residual connections[1–5] (Supplementary Figure 1) is one of the most well known architectures of Convolutional Neural Networks (CNN) used for 2D and 3D segmentation. The UNet consists of an encoder, comprising convolutional layers and downsampling layers, and a decoder offering upsampling layers (applying transpose convolution layers) and convolutional layers. The encoder-decoder structure contributed in automatically capturing information at multiple scales/resolutions. The UNet further includes skip connections, which consist of concatenated feature maps paired across the encoder and the decoder layer, to improve context and feature re-usability.
- **Fully Convolutional Network (FCN)**: The FCN architecture[6] (Supplementary Figure 2) introduced in 2017, utilizes hierarchical feature extraction with an encoder recognizing both imaging patterns and spatial information of each input image, with varying receptive fields. FCN has smaller computational requirements compared to UNet, due to the absence of the decoding module, incorporating convolution and transpose convolution operations. FCN simply upsamples the encoded features to the required output segmentations to generate masks. It hence provides faster, yet coarser, segmentations for various domains[7].
- **Inception UNet (UInc)**: The Inception module[8,9] can be used to substitute the standard convolutional block (which is a simple series of convolutional layers) of the UNet to create the UInc architecture (Supplementary Figure 3). This module describes parallel pathways of convolutional layers of different kernel sizes, to improve the representation of multi-scale features. UInc has been applied towards semantic segmentation workloads[10].
- **Spatial Decomposition Network (SDNet)**: The SDNet[11] (Supplementary Figure 4) is a well-known content-style disentanglement model for medical image segmentation. SDNet uses two different encoders to separate anatomy from appearance; a UNet encodes the anatomical information into a spatial representation and a variational autoencoder encodes the appearance into a vector one. The encoded anatomical information is represented as multi-channel binary maps of the same resolution as the input. A segmentation module is applied on the anatomy latent space to learn to predict the segmentation masks. A decoder is responsible for reconstructing the input by combining the two latent variables at multiple levels of granularity using AdaIN layers[12]. The original architecture uses FiLM layers[13] to combine these variables and a mask discriminator to support semi-supervised learning. GaNDLF currently supports the fully supervised training scheme.
- **TransUNet**: The TransUNet[14] (Supplementary Figure 5) architecture is a variant of UNet that uses a CNN-transformer hybrid encoder rather than just a CNN (UNet) or transformer (UNetR) encoder. This allows for the transformer portion of the encoder to capture long-range dependencies and global context using self-attention, while the leveraging the

resolution of the CNN feature maps. GaNDLF supports TransUNet of variable depth and will scale the number of transformer layers accordingly. GaNDLF supports the use of TransUNet on both 2D and 3D images.

- **UNetR**: The UNetR[15] (Supplementary Figure 6) architecture is a variant of UNet that uses a transformer encoder rather than the traditional CNN-based encoder. The transformer allows for capturing of long-range dependencies and global context, and it consists of a multi-head self-attention module that maps between a query and the value and key representations followed by a multilayer perceptron. GaNDLF supports UNetR of variable depth and will scale the number of transformer layers based on the size of the input image. GaNDLF supports the use of UNetR on both 2D and 3D images.
- **VGG**: The VGG[16,17] (Supplementary Figure 7) is a well-known network for performing classification and regression workloads. The original VGG has 16 convolutional layers and 3 dense layers. We have modified the final classifier layers to include a global average pooling layer followed by a single dense layer, which allows greater flexibility[18] for different types of workloads and reduce the effect of overfitting due to dense layers[19]. It is well known for its performance on the ImageNet classification challenge[20]. VGG reinforced the idea that networks should be simple and deep. VGG uses 3×3 convolution filters and 2×2 max-pooling layers with a stride of 2 throughout the architecture. The original architecture uses ReLU activation function[21] and categorical cross-entropy loss function. The initial layers of the VGG perform feature extraction and the last softmax layers act as the classifier. GaNDLF supports multiple variants of the VGG, namely, VGG11, VGG13, VGG16, VGG19, with and without batch normalization for both 2D and 3D datasets to maximize flexibility.
- **DenseNet**: The DenseNet[22] (Supplementary Figure 8) architecture is a type of convolutional neural networks that consist of dense blocks, where each layer in the block is densely connected, which is a mechanism to address the vanishing-gradient problem[23]. A unique property of the dense connections in DenseNet is that the previous layer's output and the current layer's output are concatenated instead of getting added. After the concatenation, there is a pooling layer, batch normalization, and non-linear activation layer. The DenseNet architecture can be customized based on the number of layers, and GaNDLF currently supports the DenseNet-121, DenseNet-160, DenseNet-201 and DenseNet-264 variants.
- **ResNet**: The ResNet[3] (Supplementary Figure 9) module uses shortcut connections to allow for learning of deeper architectures while avoiding the degradation of training accuracy. When the dimensions of the input and output to the block are the same, identity mapping is used, which does not introduce new parameters or increase computation complexity. If the dimensions change, linear projections are applied by the shortcut connection to match dimensions. GaNDLF supports variants of ResNet, including ResNet-18, ResNet-34, ResNet-50, ResNet-101, and ResNet-152. ResNet-18 and ResNet-34 use a pair of 3×3 convolutions for each block, while ResNet-50, ResNet-101, and ResNet-152 use a bottleneck of 1×1, 3×3, and 1×1 convolutions to reduce the number of parameters.
- **EfficientNet**: The EfficientNet[24] (Supplementary Figure 10) module uses a compound scaling method to uniformly network scale width, depth, and resolution using a set of fixed constants, which allows for networks to be scaled while achieving improved accuracy without sacrificing efficiency. The base architecture uses mobile inverted bottleneck convolutions (MBConv) with squeeze-and-excitation optimization, which increases efficiency by the use of a narrow to wide to narrow approach rather than the wide to narrow to wide approach of residual blocks. GaNDLF supports multiple variants of EfficientNet, from EfficientNetB0 through EfficientNetB7.
- **ImageNet-trained 2D models**: GaNDLF also provides functionality of transfer learning based on popular architectures pre-trained on the ImageNet data[20]. Every architecture's first and last layers are modified to be able to process input images of any size, and only output the relevant number of classes for each problem, respectively. The rest of the layers retain weights from the ImageNet data. This allows for more efficient training, with a potential for better convergence result[25].

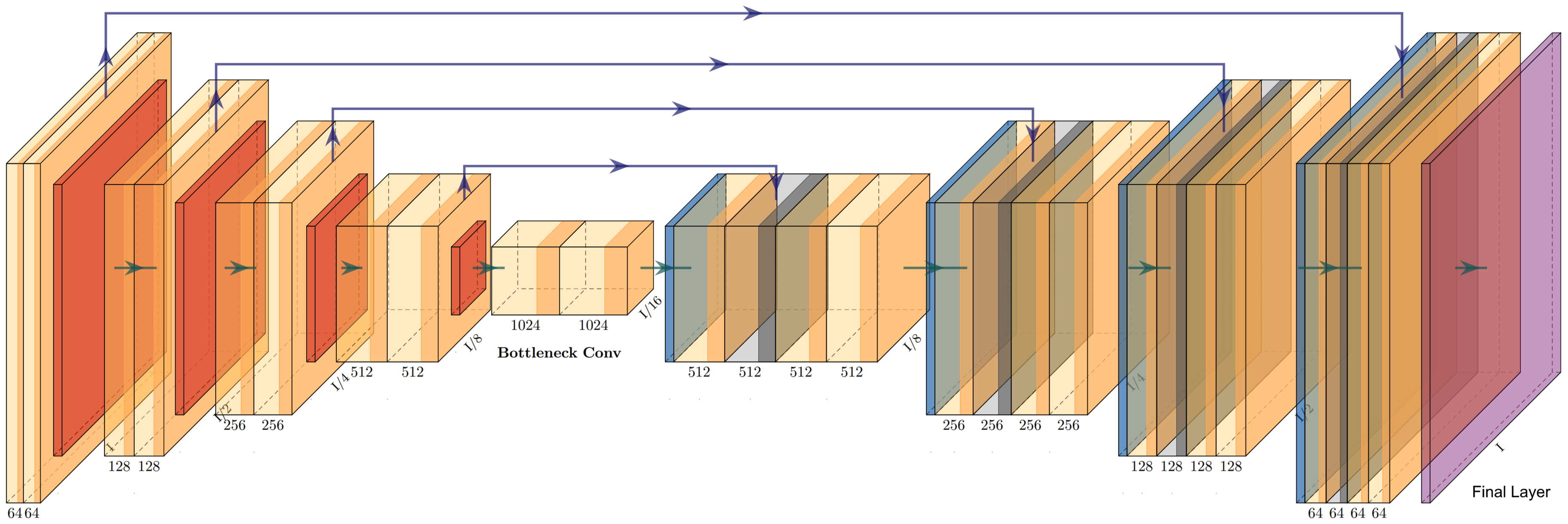

Supplementary Figure 1: UNet, incorporated in GaNDLF with and without residual connections, is a well-known architecture for segmentation.

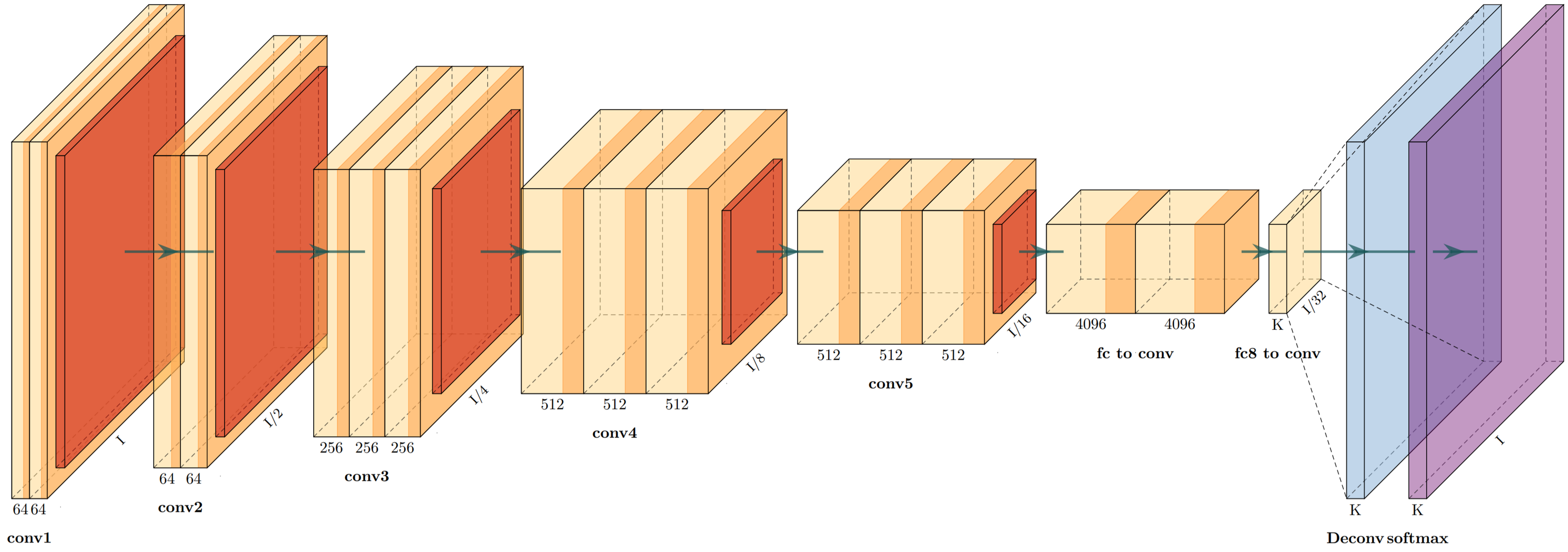

Supplementary Figure 2: Fully Convolutional Network.

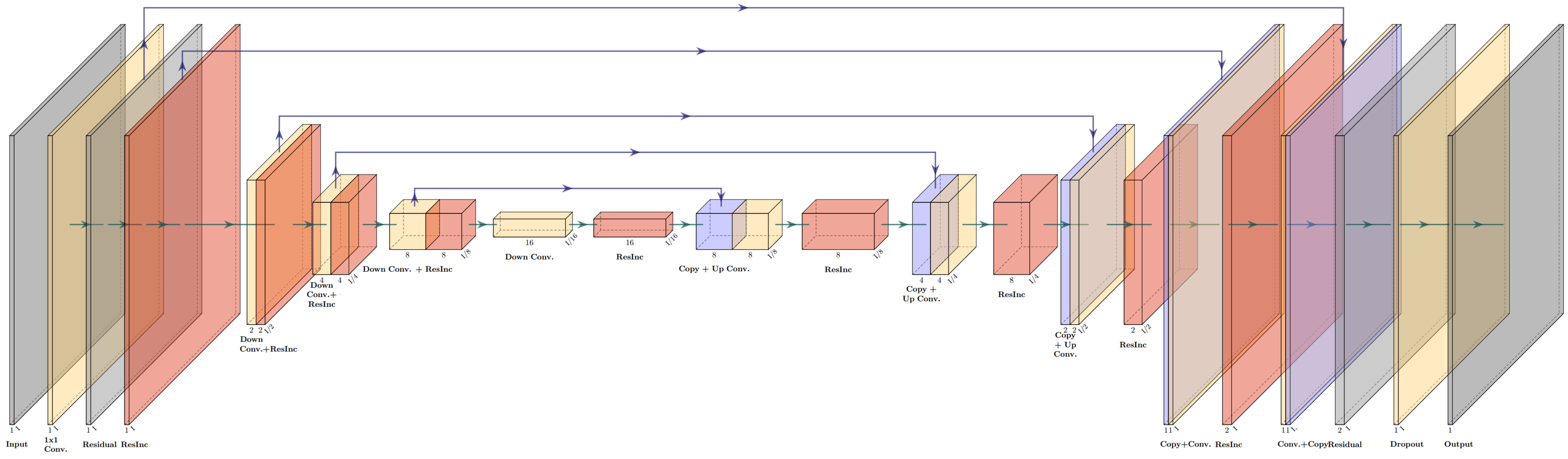

Supplementary Figure 3: Inception UNet, which incorporates inception blocks in a UNet.

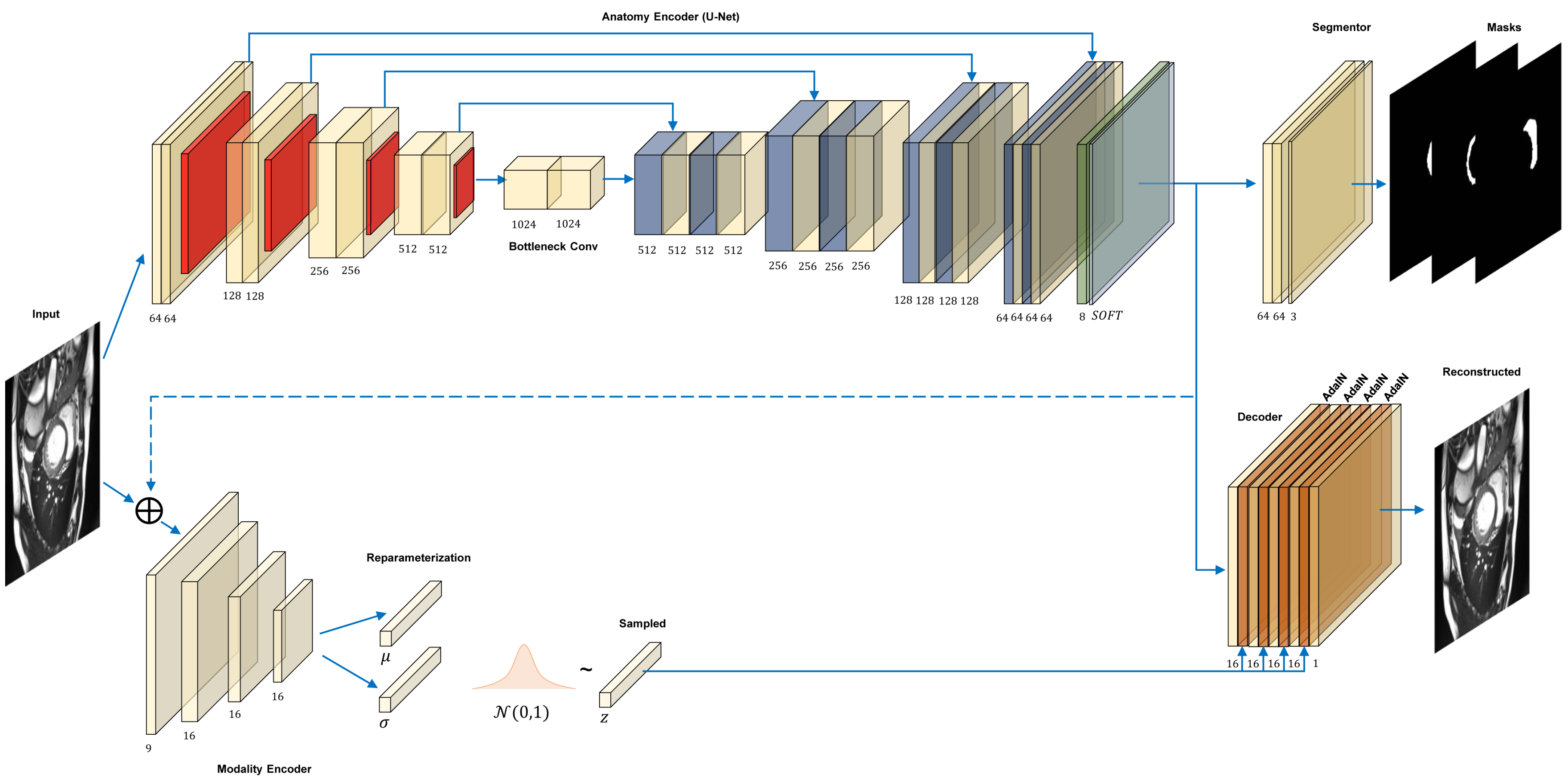

Supplementary Figure 4: SDNet, a unique architecture incorporating concept of disentangled learning for segmentation.

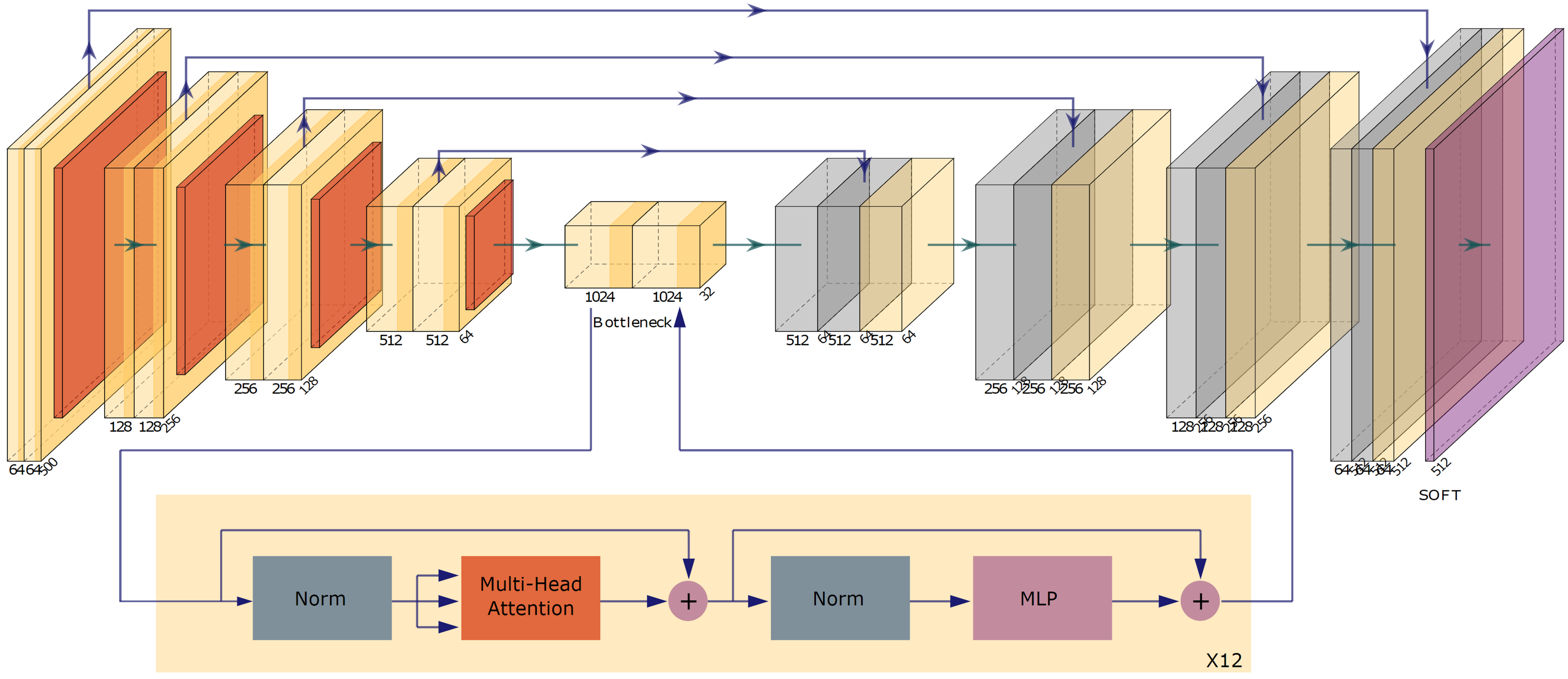

Supplementary Figure 5: TransUNet, a state-of-the-art segmentation architecture incorporate vision transformers.

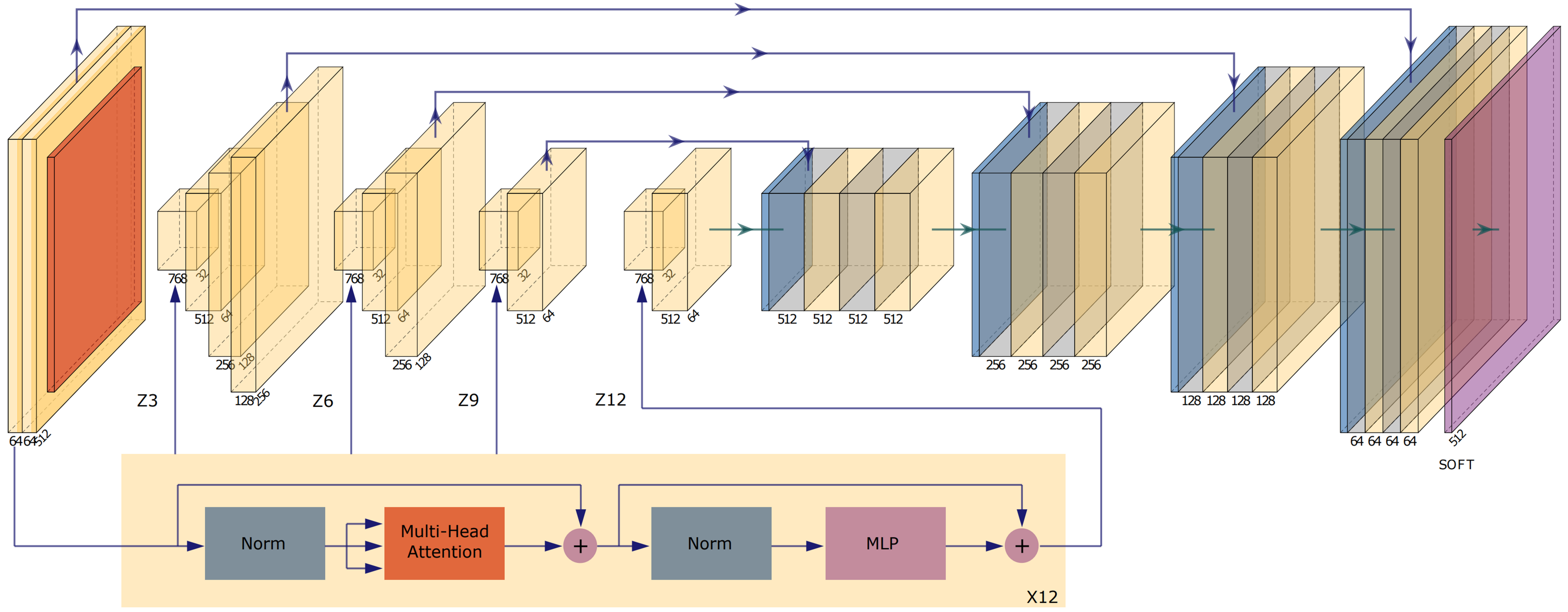

Supplementary Figure 6: UNetR, a state-of-the-art segmentation architecture incorporate vision transformers..

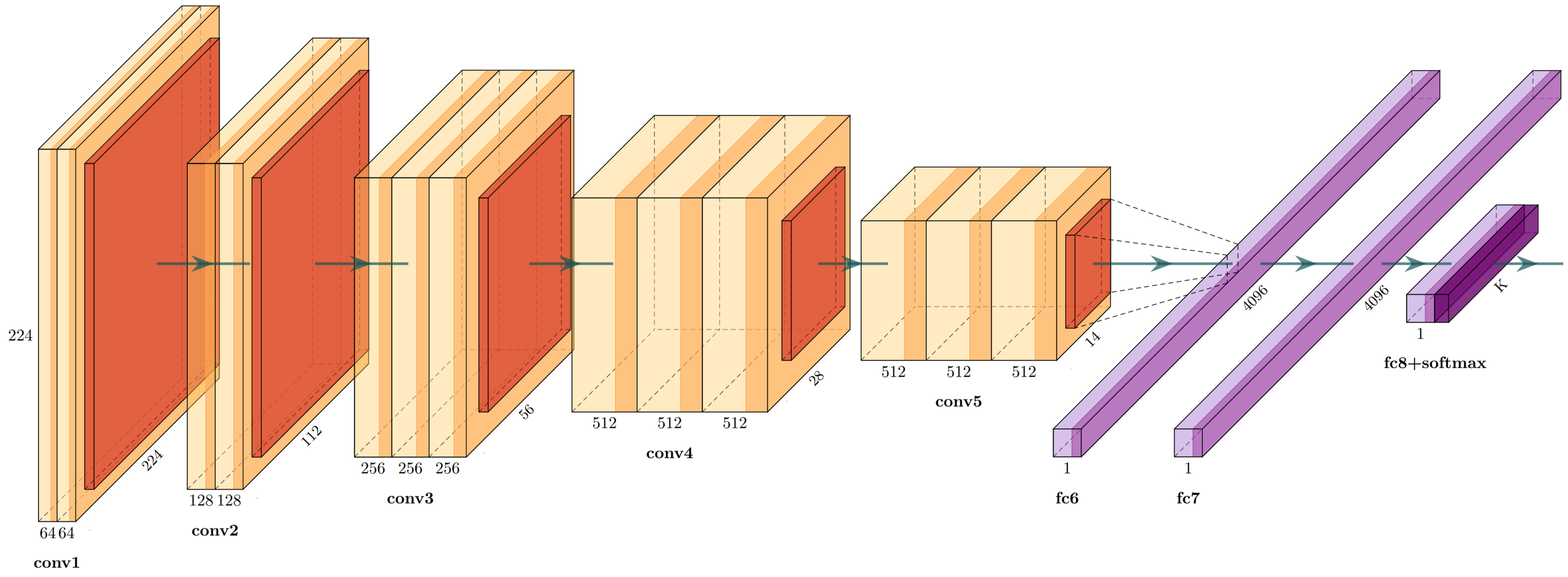

Supplementary Figure 7: VGG16, a well-known architecture for tackling regression and classification tasks.

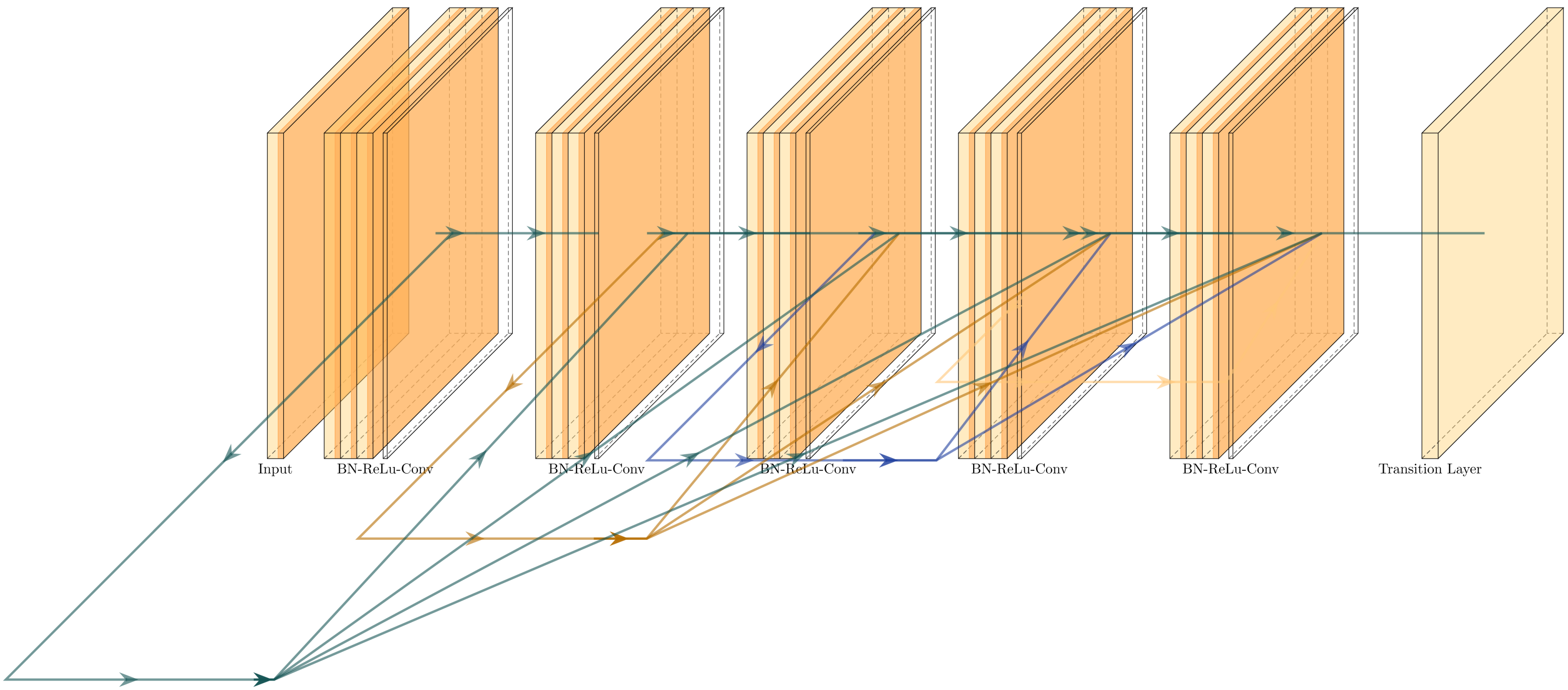

Supplementary Figure 8: DenseNet, a well-known architecture for tackling regression and classification tasks.

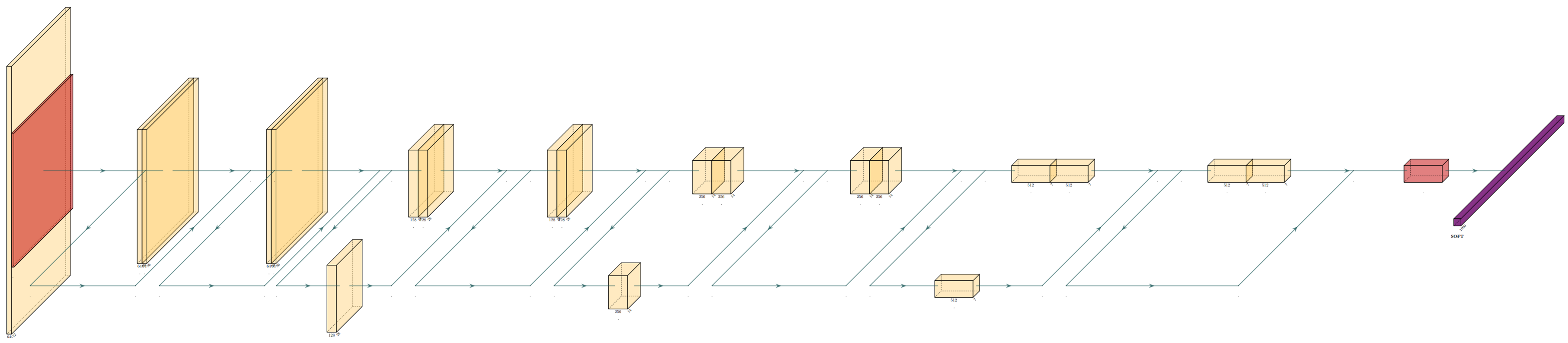

Supplementary Figure 9: ResNet, a well-known state-of-the-art architecture for tackling regression and classification tasks.

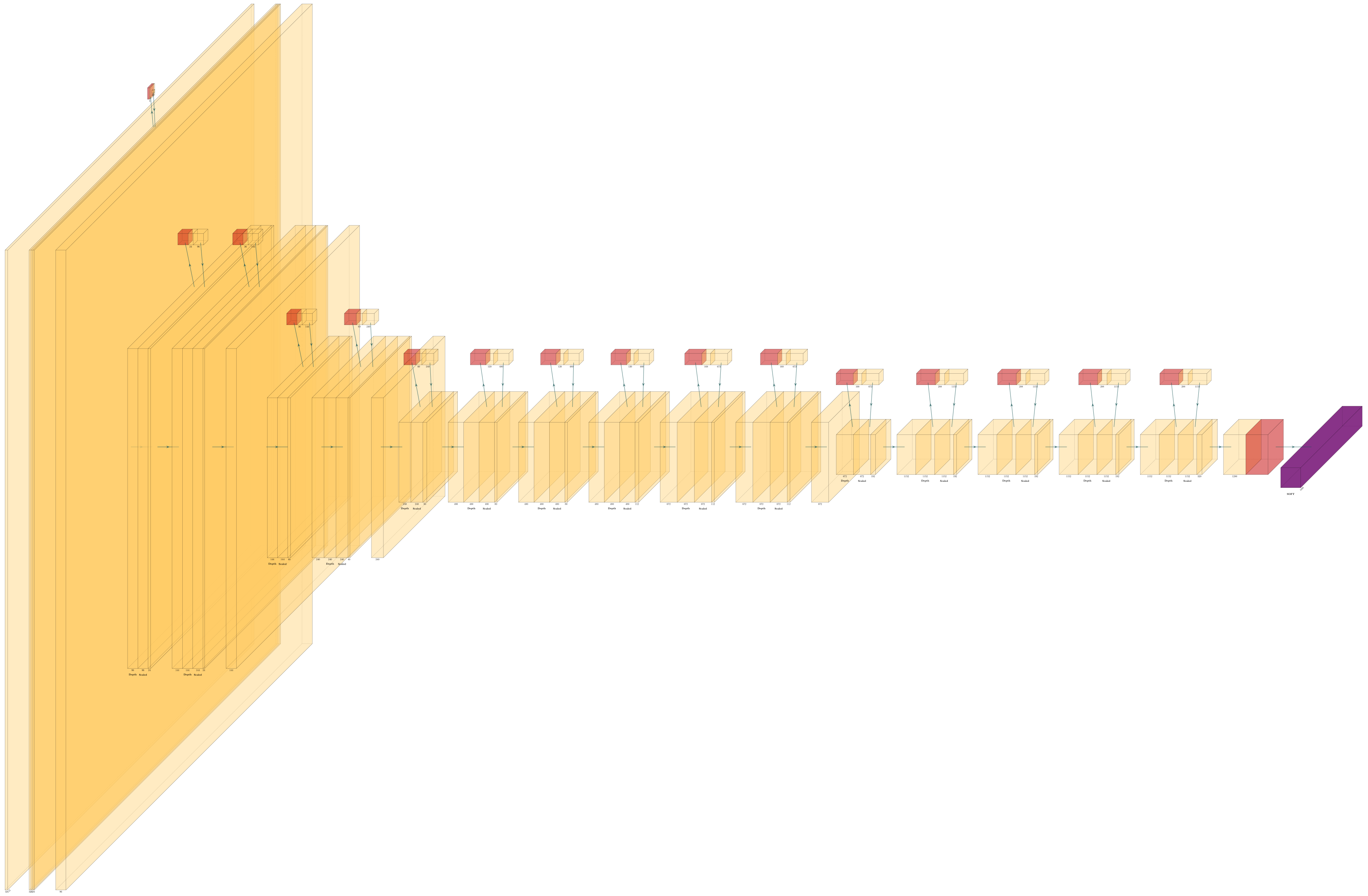

Supplementary Figure 10: EfficientNet, a well-known state-of-the-art architecture for tackling regression and classification tasks.

Supplementary References

[1] Olaf Ronneberger, Philipp Fischer, and Thomas Brox. “U-net: Convolutional networks for biomedical image segmentation”. In: International Conference on Medical image Springer. 2015, pp. 234–241. DOI: 10.1007/978-3-319-24574-4_28.

[2] Michal Drozdzal et al. “The importance of skip connections in biomedical image segmentation”. In: Deep Learning and Data Labeling for Medical Applications. Springer, 2016, pp. 179–187. DOI: 10.1007/978-3-319-46976-8_19.

[3] Kaiming He et al. “Deep residual learning for image recognition”. In: Proceedings of the IEEE conference on computer vision and pattern recognition. 2016, pp. 770–778. DOI: 10.1109/CVPR.2016.90.

[4] Özgün Çiçek et al. “3D U-Net: learning dense volumetric segmentation from sparse annotation”. In: International conference on medical image computing and computer-a Springer. 2016, pp. 424–432. DOI: 10.1007/978-3-319-46723-8_49.

[5] Siddhesh Thakur et al. “Brain extraction on MRI scans in presence of diffuse glioma: Multi-institutional performance evaluation of deep learning methods and robust modality-agnostic training”. In: NeuroImage 220 (2020), p. 117081. DOI: 10.1016/j.neuroimage.2020.117081.

[6] Jonathan Long, Evan Shelhamer, and Trevor Darrell. “Fully convolutional networks for semantic segmentation”. In: Proceedings of the IEEE conference on computer visio 2015, pp. 3431–3440. DOI: 10.1109/CVPR.2015.7298965.

[7] Geert Litjens et al. “A survey on deep learning in medical image analysis”. In: Medical image analysis 42 (2017), pp. 60–88. DOI: 10.1016/j.media.2017.07.005.

[8] Christian Szegedy et al. “Going deeper with convolutions”. In: Proceedings of the IEEE conference on computer vision and pattern recognition. 2015, pp. 1–9. DOI: 10.1109/CVPR.2015.7298594.

[9] Christian Szegedy et al. “Inception-v4, inception-resnet and the impact of residual connections on learning”. In: Proceedings of the AAAI Conference on Artificial Intellige Vol. 31. 2017, pp. 4278–4284. DOI: 10.48550/arXiv.1602.07261.

[10] Jimit Doshi et al. “DeepMRSeg: a convolutional deep neural network for anatomy and abnormality segmentation on MR images”. In: arXiv preprint arXiv:1907.02110 (2019). DOI: 10.48550/arXiv.1907.02110.

[11] Agisilaos Chartsias et al. “Disentangled representation learning in cardiac image analysis”. In: Medical image analysis 58 (2019), p. 101535. DOI: 10.1016/j.media.2019.101535.

[12] Xun Huang and Serge Belongie. “Arbitrary style transfer in real-time with adaptive instance normalization”. In: Proceedings of the IEEE International Conference on Com 2017, pp. 1501–1510. DOI: 10.1109/ICCV.2017.167.

[13] Ethan Perez et al. “Film: Visual reasoning with a general conditioning layer”. In: Proceedings of the AAAI Conference on Artificial Intelligence. Vol. 32. 2018, p. 1. DOI: 10.48550/arXiv.1709.07871.

[14] Jieneng Chen et al. “Transunet: Transformers make strong encoders for medical image segmentation”. In: arXiv preprint arXiv:2102.04306 (2021). DOI: 10.48550/arXiv.2102.04306.

[15] Ali Hatamizadeh et al. “Unetr: Transformers for 3d medical image segmentation”. In: Proceedings of the IEEE/CVF Winter Conference on Applications of Computer Visi 2022, pp. 574–584. DOI: 10.48550/arXiv.2103.10504.

[16] Karen Simonyan and Andrew Zisserman. “Very deep convolutional networks for large-scale image recognition”. In: arXiv preprint arXiv:1409.1556 (2014). DOI: 10.48550/arXiv.1409.1556.

[17] Avi Ben-Cohen et al. “Fully convolutional network for liver segmentation and lesions detection”. In: Deep learning and data labeling for medical applications. Springer, 2016, pp. 77–85. DOI: 10.1007/978-3-319-46976-8_9.

[18] Ting-Yun Hsiao et al. “Filter-based deep-compression with global average pooling for convolutional networks”. In: Journal of Systems Architecture 95 (2019), pp. 9–18. DOI: 10.1016/J.SYSARC.2019.02.008.

[19] Min Lin, Qiang Chen, and Shuicheng Yan. “Network In Network”. In: (2013). DOI: 10.48550/ARXIV.1312.4400. URL: https://arxiv.org/abs/1312.4400.

[20] Jia Deng et al. “Imagenet: A large-scale hierarchical image database”. In: 2009 IEEE conference on computer vision and pattern recognition. Ieee. 2009, pp. 248–255. DOI: 10.1109/CVPR.2009.5206848.

[21] Abien Fred Agarap. “Deep learning using rectified linear units (relu)”. In: arXiv preprint arXiv:1803.08375 (2018). DOI: 10.48550/arXiv.1803.08375.

[22] Gao Huang et al. “Densely connected convolutional networks”. In: Proceedings of the IEEE conference on computer vision and pattern recognition. 2017, pp. 4700–4708. DOI: 10.1109/CVPR.2017.243.

[23] Sepp Hochreiter. “The vanishing gradient problem during learning recurrent neural nets and problem solutions”. In: International Journal of Uncertainty, Fuzziness and Kn 6.02 (1998), pp. 107–116. DOI: 10.1142/S0218488598000094.

[24] Mingxing Tan and Quoc Le. “Efficientnet: Rethinking model scaling for convolutional neural networks”. In: International conference on machine learning. PMLR. 2019, pp. 6105–6114. DOI: 10.48550/arXiv.1905.11946.

[25] Simon Kornblith, Jonathon Shlens, and Quoc V Le. “Do better imagenet models transfer better?” In: Proceedings of the IEEE/CVF conference on computer vision and patt 2019, pp. 2661–2671. DOI: 10.1109/CVPR.2019.00277.
