# Supplementary material for: GaNDLF: the generally nuanced deep learning framework for scalable end-to-end clinical workflows
Source: Commun Eng. 2023 May 16;2:23. doi: 10.1038/s44172-023-00066-3 (PMC10956028; doi:10.1038/s44172-023-00066-3)
Supplement: Supplementary file 3 — Reporting Summary [file 44172_2023_66_MOESM3_ESM.pdf]

nature portfolio

Corresponding author(s): Spyridon Bakas; sbakas@upenn.edu

Last updated by author(s): Feb 20, 2023

Reporting Summary

Nature Portfolio wishes to improve the reproducibility of the work that we publish. This form provides structure for consistency and transparency in reporting. For further information on Nature Portfolio policies, see our Editorial Policies and the Editorial Policy Checklist.

Statistics

For all statistical analyses, confirm that the following items are present in the figure legend, table legend, main text, or Methods section.

n/a	Confirmed	
☐	☒	The exact sample size (n) for each experimental group/condition, given as a discrete number and unit of measurement
☒	☐	A statement on whether measurements were taken from distinct samples or whether the same sample was measured repeatedly
☐	☒	The statistical test(s) used AND whether they are one- or two-sided<br>*Only common tests should be described solely by name; describe more complex techniques in the Methods section.*
☐	☒	A description of all covariates tested
☐	☒	A description of any assumptions or corrections, such as tests of normality and adjustment for multiple comparisons
☐	☒	A full description of the statistical parameters including central tendency (e.g. means) or other basic estimates (e.g. regression coefficient) AND variation (e.g. standard deviation) or associated estimates of uncertainty (e.g. confidence intervals)
☐	☒	For null hypothesis testing, the test statistic (e.g. F, t, r) with confidence intervals, effect sizes, degrees of freedom and P value noted<br>*Give P values as exact values whenever suitable.*
☒	☐	For Bayesian analysis, information on the choice of priors and Markov chain Monte Carlo settings
☒	☐	For hierarchical and complex designs, identification of the appropriate level for tests and full reporting of outcomes
☐	☒	Estimates of effect sizes (e.g. Cohen's d, Pearson's r), indicating how they were calculated

Our web collection on statistics for biologists contains articles on many of the points above.

Software and code

Policy information about availability of computer code

Data collection	We used a combination of privately and publicly held datasets in this study, and all related descriptions can be found in the "Data availability" section of the manuscript. Each dataset is processed using a different mechanism (which is dependent on the either the dataset itself or the application at hand), and we have that visibility for the public datasets, and these are noted in the "Methods" section.
Data analysis	All data was processed using the Generally Nuanced Deep Learning Framework (GaNDLF), and the source code can be found at: github.com/mlcommons/GaNDLF

For manuscripts utilizing custom algorithms or software that are central to the research but not yet described in published literature, software must be made available to editors and reviewers. We strongly encourage code deposition in a community repository (e.g. GitHub). See the Nature Portfolio guidelines for submitting code & software for further information.

Data

Policy information about availability of data

All manuscripts must include a data availability statement. This statement should provide the following information, where applicable:

- Accession codes, unique identifiers, or web links for publicly available datasets
- A description of any restrictions on data availability
- For clinical datasets or third party data, please ensure that the statement adheres to our policy

Provide your data availability statement here.

Human research participants

Policy information about studies involving human research participants and Sex and Gender in Research.

Reporting on sex and gender	This study does not refer to sex or gender anywhere in the manuscript.
Population characteristics	Population characteristics are different per dataset being used, and wherever present, have been described in the "Methods" section of the manuscript.
Recruitment	All results reported were from retrospectively collected datasets, and explicit permission to use data for research purposes was obtained by the data collector/collection agency in question.
Ethics oversight	Since multiple datasets were used, approvals from different institutional review boards were used.

Note that full information on the approval of the study protocol must also be provided in the manuscript.

Field-specific reporting

Please select the one below that is the best fit for your research. If you are not sure, read the appropriate sections before making your selection.

☒ Life sciences ☐ Behavioural & social sciences ☐ Ecological, evolutionary & environmental sciences

For a reference copy of the document with all sections, see nature.com/documents/nr-reporting-summary-flat.pdf

Life sciences study design

All studies must disclose on these points even when the disclosure is negative.

Sample size

- Segmentation of Brain in MRI: 2520 MRI scans.
- Segmentation of Brain Tumor Sub-regions in MRI: 371 multi-modal MRI scans.
- Whole Brain Parcellation in MRI: 30 MRI scans.
- Segmentation of Breast Tissue using DBT: 1080 digital tomosynthesis scans.
- Segmentation of Structural Tumor Volume Breast MRI: 163 subjects with multiple timepoints representing pre-, maximal, and post-maximal excitation phases of the injected contrast agent.
- Segmentation of Lung Field in CT: 1173 low-dose CT scans of patients having lung cancer (500) and COVID-19 (673).
- Segmentation of Retinal Fundus: 400 images.
- Segmentation of Quadrants in Panoramic Dental X-Ray Images: 900 panoramic x-ray images.
- Segmentation of Colorectal Cancer in WSI: 180000 independent patches for training, 30000 independent patches for validation, and 30 whole slide images for testing phase.
- Brain Age Prediction from MRI: The data used was from the UK Biobank169 and a multisite schizophrenia consortium.
- Prediction of the EGFRvIII mutation in brain tumors using structural mpMRI: The data used in this study is not publicly available due to the restrictions from the acquiring institution. These restrictions also prevent the data from being made available upon request from the authors.
- Classification of Diabetic Foot Ulcer Images: The data used was from the Diabetic Foot Ulcer Grand Challenge (DFUC) of 2021.
- Classification of Tumor Infiltrating Lymphocytes: The data used is available in The Cancer Genome Atlas (TCGA).

Data exclusions	Each application had its own exclusion/inclusion criteria. All available data was included for all applications with the exception of "Segmentation of Structural Tumor Volume in Breast MRI", where data from 74 subjects were excluded based on unavailability of all required timepoints, and this criteria was previously established in the study.
Replication	All configurations and code will be made available on GaNDLF's GitHub repository [github.com/mlcommons/GaNDLF] following the publication of the manuscript.
Randomization	Most applications performed randomized k-fold training where the entire cohort was iteratively split into training, validation, and testing sets, and then the performance analysis was done. For the applications where these sets were previously defined in the dataset, the same split information was used.
Blinding	N.A.

Reporting for specific materials, systems and methods

We require information from authors about some types of materials, experimental systems and methods used in many studies. Here, indicate whether each material, system or method listed is relevant to your study. If you are not sure if a list item applies to your research, read the appropriate section before selecting a response.

Materials & experimental systems

n/a	Involved in the study
☒	☐ Antibodies
☒	☐ Eukaryotic cell lines
☒	☐ Palaeontology and archaeology
☒	☐ Animals and other organisms
☒	☐ Clinical data
☒	☐ Dual use research of concern

Methods

n/a	Involved in the study
☒	☐ ChIP-seq
☒	☐ Flow cytometry
☐	☒ MRI-based neuroimaging

Magnetic resonance imaging

Experimental design

Design type: N.A. (we only used retrospective data).

Design specifications: N.A. (we only used retrospective data).

Behavioral performance measures: N.A. (we only used retrospective data).

Acquisition

Imaging type(s): Structural

Field strength: 1.5 to 3T

Sequence & imaging parameters: For brain images: T1, T2, T1-post contrast, and T2-weighted FLAIR
For breast images: T1-weighted pre- and 2 post-contrast images.

Area of acquisition: Brain and breast.

Diffusion MRI: ☐ Used ☒ Not used

Preprocessing

Preprocessing software: The Cancer Imaging Phenomics Toolki [github.com/CBICA/CaPTk] version 1.8.0 to 1.9.1 was used for preprocessing.

Normalization: Normalization was done by z-scoring.

Normalization template: The SRI 24 atlas [www.nitrc.org/projects/sri24] was used to normalize the resolution and size of brain images.

Noise and artifact removal: N.A.

Volume censoring: N.A.

Statistical modeling & inference

Model type and settings: N.A.

Effect(s) tested: N.A.

Specify type of analysis: ☐ Whole brain ☐ ROI-based ☒ Both

Anatomical location(s): Whole brain, breast, and lung

Statistic type for inference (See Eklund et al. 2016): N.A.

Correction: N.A.

Models & analysis

n/a	Involved in the study
☒	☐ Functional and/or effective connectivity
☒	☐ Graph analysis
☒	☐ Multivariate modeling or predictive analysis
